# Indications for replantation and factors that predict success

**DOI:** 10.1007/s00590-023-03671-2

**Published:** 2023-08-18

**Authors:** Dana Bregman, Luke Nicholson

**Affiliations:** Department of Orthopedics, University of Southern California, Los Angeles, California USA

**Keywords:** Replantation, Revascularization, Microsurgery, Hand surgery, Indications, Contraindications

## Abstract

Since the advent of replantation techniques, there has been uneven progress in terms of success—even considering whether success is defined as continued perfusion of the digit or long term functional outcomes. Early enthusiasm and success have not been maintained despite increasing familiarity with microsurgical techniques and greater understanding of how to optimize outcomes for the individual components of replantation such as tendon repair, nerve repair, and osteosynthesis. Practitioners participating in the care of patients undergoing replantation should be familiar with the history and current understanding of both the indications and contraindications for the procedure, post-operative rehabilitation, and factors contributing to functional outcomes. A review of the literature from the first publications on the topic demonstrates evolution in practice and understanding of which patients should be indicated for replantation. Indications can be considered in three broad categories: injury factors, patient factors, and care context factors. These factors intersect with one another and can inform the surgeon pre-operatively regarding the most likely outcome for a given patient. This insight is critical to discuss pre-operatively with the patient in order to make a shared decision about how to manage their injury.

## Introduction

The development of microsurgical tools and techniques in the twentieth century enabled hand surgeons to successfully restore bodily integrity to those who had suffered traumatic amputations. Pioneers in this field include Drs. Ronald Malt and Charles McKhann who demonstrated the feasibility of replanting transhumeral amputations in 1964, as well as Drs. Kamatsu and Tamai who replanted a thumb amputated at the metacarpophalangeal joint in 1965 [1, 2]. The next few decades were marked by continued refinements to technique, including the enumeration of what injury-related factors are critical to digit survival [3, 4]. Reported success rates in the early era of replantation were impressively high, but these rates have not been consistently maintained [5, 6]. It is paramount to note that success itself is a complex metric to define in the context of replantation. Although replanted digits may be considered “successful” if they maintain adequate arterial perfusion and venous drainage, is this the most salient outcome for a patient who is left with a finger that is stiff, painful, and insensate? There is a relative paucity of literature on functional outcomes although more recent studies have attempted to address this deficit [7, 8].

The indications and contraindications to replantation deserve to be considered in the context of declining rates of survival in digit replantation in the USA. Narrower definitions of what constitutes a replantable digit will contribute to higher survival rates as the denominator of this ratio gets smaller. It is possible that as indications for replantation narrowed from the first replantations to the 1990s, this led to the superior outcomes in terms of digit survival. However, other data suggest that the surgeons with the highest survival rates for replantation are those with the highest surgical volumes, who are also more likely to attempt replantation than lower-volume surgeons [9]. This problematizes the notion that high reported success rates are attributable to surgeons electing to attempt replantation under only the most favorable of circumstances.

A thorough discussion of the contributors to the decline in both replantation attempts and survival in the last thirty years in the USA is beyond the scope of this paper. Various authors have implicated a decrease in surgeon enthusiasm for replantation due to lack of procedural experience, an increased awareness of poor functional outcomes, and low reimbursement rates relative to the complexity of the procedure [9, 10]. This may lead to a self-perpetuating cycle in which infrequent replantation attempts lead to lack of experience and surgical confidence, resulting in poor surgical skills, which engenders the perception that successful functional outcomes are rare and therefore, further limits replantation attempts. This fatalistic attitude deserves to be challenged. An awareness of historically superior survival rates should lead any hand surgeon to question what we can do better to advance replantation surgery in the twenty-first century.

Early published indications for replantation focused primarily on injury-related factors (Table 1) and noted that replantation should always be attempted in children. More recently, increased attention has focused on patient-related factors that may impede success [11, 12]. Furthermore, some of the historical contraindications to replantation, such as a digit amputated proximal to the insertion of the flexor digitorum superficialis (FDS), or an index finger, have been challenged by more recent studies that include functional outcomes [13, 14]. The following discussion of indications for replantation is based on, when possible, a consideration of success defined as the best functional outcome for the patient. In all operative procedures, but perhaps especially in the context of replantation and revascularization, the influence of the surgeon’s biases and ego are particularly relevant. The opportunity to restore perfusion to a dysvascular or amputated part is compelling as it rewards the surgeon with immediate gratification and validation of his or her abilities. The honor of success and the recognition it may bring is meaningless if the perspective of the patient is forgotten. Data on this subject continue to accrue although there are several subtopics for which there does not exist good evidence.

**Table 1:** Summary of indications for replantation from 1978 to 1981 [3, 4]

Indications	Contraindications
Thumb	Crushed or mutilated parts
Multiple digits	Single digit proximal to insertion of flexor digitorum profundus
Patient is a child	Prolonged warm ischemia time (> 6–8 h; ischemia time based on amputation level not specified)
Amputation through palm or proximal	Patient with other life-threatening injuries or significant systemic disease

Although prior publications have focused on injury and patient factors pertinent to the likelihood of replantation survival, few have focused on the “care context”. It is known that high-volume replantation centers have higher success rates, but the granular forces which contribute to this finding have not been parsed [15]. The care context includes variables related to the surgeon, the operative team, the operating room (OR) and hospital resources, post-operative care including monitoring and ability to return to the OR when needed, and peri-operative therapeutics such as regional anesthesia and anticoagulation protocols. These three considerations; that is, the injury, the patient, and the care context; intersect and mutually influence the likelihood of achieving a viable and functional extremity. Therefore, we posit that they are all pertinent to the discussion of indications and contraindications for replantation.

### Patient factors

Patient age is a factor with one of the greatest influences on the decision to replant. Pediatric amputations are fortunately relatively rare injuries and are most common in older children (ages 15–19). Data on the long-term outcomes following pediatric digit replantation are limited but indicate that children generally regain good function and sensation following replantation [16]. However, the initial replantation survival rates in children are lower than those in adults; this can be attributed to more aggressive attempts for replantation in this population, increased technical difficulty in repairing smaller structures, and greater risk of vasospasm in children [17]. Children undergoing replantation are less likely to have a post-operative complication, require an eventual amputation, or have a prolonged hospital stay compared to adults [18]. Replantation in elderly patients has not historically been performed as enthusiastically due to concerns for co-morbid conditions precluding prolonged anesthesia, the increased prevalence of atherosclerosis, and the perception that elderly patients have lower functional demands. Digit replantation in older patients up to the age of 70 has been shown to have good survival; outcomes for those above this age are not as promising, although these findings are inconsistent [11, 19]. The premorbid functional status of an older patient being assessed as a candidate for replantation is more informative than their chronologic age.

Other patient factors have a significant influence on replantation success. Hustedt et al. reviewed data on over 11,700 replantations and identified the co-morbid conditions most associated with replant failure. These include psychotic disorders, peripheral vascular disease, electrolyte imbalance, depression, anemia, obesity, alcohol abuse, and tobacco use. Furthermore, when patients have 3 or more co-morbidities, the relative risk of replant failure increases significantly, along with the risk of post-operative complications and hospital cost. Patients with high-risk co-morbidities, or those with 3 or more co-morbidities, should be considered carefully prior to attempting replantation [11].

Of greater immediate significance compared to non-acute co-morbid conditions are life-threatening injuries that preclude prolonged surgery; in this scenario, it may be appropriate to consider ectopic banking or extracorporeal membrane oxygenation (ECMO) of the amputated part. This strategy can also be used when there is severe contamination or multilevel injury proximal to the amputation that requires serial debridement or soft tissue coverage prior to replantation [20].

Patient factors that influence success of replantation extend beyond medical diagnoses and demographics. A patient’s occupation, hobbies, belief structures, support system, and motivations greatly influence the person’s candidacy for replantation as well as likelihood for achieving a functional outcome. Social support for the patient has not been widely studied with regards to its influence on the success of the replanted part; however, there is evidence that patients who receive support in the form of positive psychological suggestions have improved overall function and mood [21]. It has also been found that patients with higher preoperative anxiety have a higher risk of replant failure [22]. It may be advantageous to the surgeon to include in the discussion of risks and benefits of replantation those individuals the patient identifies as being responsible for care and support post-operatively.

Patients presenting with an amputation should be counseled prior to undergoing the initial replantation surgery that a successful functional result often requires secondary and tertiary revision procedures to optimize the function of the part [23, 24]. These procedures often are performed 4–6 months following the initial injury and are dependent on high quality hand therapy and rehabilitation for success. These procedures consist of extensor and flexor tenolysis, as well as possible joint contracture release. Some patients may require secondary tendon reconstruction, or surgery to address nonunion or malunion. Finally, procedures for soft tissue contracture or neuroma management may also be required. The order and extent of these procedures depends on the specific nature of the deficits and recommendations for sequencing vary throughout the literature [25]. The operative surgeon should preemptively inform the patient of the potential need for these additional procedures and rehabilitation to achieve a mobile, sensate, and useful replanted part.

When discussing the risks and benefits of replantation with a patient, the surgeon should elicit the patient’s premorbid functional demands as well as their need to return to work. If a patient expresses a need to return to work immediately and has an amputation that is of borderline indication for replantation, the reality of the demands of rehabilitation should be part of a frank discussion. Conversely, in amputated parts that are injured so severely as to preclude a highly functional outcome, it is important to also consider the patient’s personal and cultural attitudes toward bodily integrity and aesthetics [26].

Patient factors important to consider include beliefs and attitudes around elements of care that may be required for successful replantation. Patients should be counseled on possible blood transfusion, expected length of stay, the potential need to return to the OR, the need to use autologous tissue grafts from non-injured extremities, and the need for salvage interventions including leeches. While not every patient will require these measures, they should be discussed early to ensure the patient understands what may be required to achieve a successful replantation and to inform the surgeon of the patient’s candidacy for replantation.

The above discussion emphasizes a shared decision model of informed consent; this has been advocated by other authors in the context of counseling patients with borderline indications for replantation [27]. This raises the question: to what extent can a patient who has just sustained an injury resulting in both a physical wound as well as a traumatic assault to their sense of bodily integrity make an informed and rational decision? Often informed consent is a process that is rushed in the setting of trauma when the surgery is time-sensitive. The surgeon should strive to elucidate whatever discrepancies exist between the likely outcome and the patient’s stated expectations preoperatively and address them directly [28].

### Injury factors

The most frequently discussed topic within the literature on indications and contraindications for replantation is injury factors. The first consideration is the location of the injury in terms of which digit, which level within a digit, and more proximal amputations. Giladi and colleagues investigated the functional disability of digit amputees at different levels and found that amputation level alone does not predict patient reported functional outcomes [29]. Given that the thumb is responsible for a plurality of hand function, replantation of a proximally amputated thumb should almost always be attempted when feasible. In addition, when a patient presents with multiple digit amputations, the resulting defect would profoundly affect hand function and therefore, replantation is typically recommended and attempted. Traditional teaching advises surgeons to avoid replantation of digits amputated proximal to the insertion of the FDS as this was associated with significant loss of motion at the proximal interphalangeal joint [30]. However, more recent studies that investigated patient reported outcomes found favorable results with replantations at this level despite the decrease in range of motion [13].

A study of over 1000 patients with amputations at various Tamai levels (Table 2) who underwent either attempted replantation or amputation examined Michigan Hand Outcome Questionnaire (MHQ) values to assess the cost and utility of replantation versus amputation in different injuries. They found that replantation of small fingers at any level, ring fingers at Tamai I–III levels, and long fingers at Tamai level I were all relative contraindications to replantation as the patient did not have additional benefit of replantation compared to revision amputation. All other injury levels did show benefit [14]. This supports performing replantations proximal to the FDS insertion as well as replantation of index fingers, both of which are still controversial in many centers.

**Table 2:** Tamai levels of amputation

Tamai level	Description
I	Distal to the flexor digitorum profundus (FDP) or flexor pollicis longus (FPL) insertion
II	Distal interphalangeal joint to the FDP insertion or interphalageal joint to the FPL insertion
III	Middle phalanx distal to FDS insertion or proximal phalanx distal to flexor pollicis brevis insertion
IV	Proximal phalanx to middle phalanx FDS insertion
V	Metacarpophalangeal joint and proximal

Transmetacarpal amputation patients often have good functional outcomes with a high rate of returning to work [31]. More proximal replantations such as within the carpus, at the radiocarpal joint, or in the forearm are more likely to remain perfused, but also more likely to have diminished total active motion (TAM) compared to more distal replantations [32]. The outcome data are limited but suggests that replantation at these levels still provides functional benefit to the patient [33].

An important consideration is the presence of muscle within the part and the effect of ischemia time. For digits, the accepted limits are 6–12 h of warm ischemia time and 12–24 h of cold ischemia time, although there are case reports of success replanting digits with far greater duration of ischemia. For major limb replantations, success decreases after 2–4 h of warm ischemia time and 6–8 h of cold ischemia time [34].

The mechanism of injury has a significant impact on the success of replantation. Success is greatest in those amputations due to a sharp, “guillotine” type injury. This is not often the case. On occasion, an injury can appear to have been sustained by a sharp mechanism but is actually the result of a high force crush mechanism with a greater zone of injury than initially assumed. Blunt injuries and crush injuries have worse outcomes compared to sharp injuries [35]. Avulsion injuries have worse outcomes both in terms of viability as well as TAM. The use of vein grafts can improve vascular viability in these injuries [36].

Consideration should also be made of the patient’s other injuries. The outcomes of non-vascular injuries to adjacent digits that require rehabilitation may be significantly impacted by the decision to replant a neighboring digit [12]. A patient may present with pre-existing limited function of other digits or prior amputations, increasing the relative value of the replantation attempt. Injury in other parts of the body may also have an impact on the feasibility of a replantation. For example, a patient with prior lower extremity injuries may not have available donor material in that location for vein or nerve reconstruction.

The broad range of injury-specific variables preclude the development of a straight-forward enumeration of indications and contraindications. These injury-specific considerations intersect as well with patient-specific factors that may influence a surgeon’s clinical judgment regarding the feasibility of obtaining a good outcome. There is yet more complexity to consider when assessing the reasonableness of offering a patient a replantation attempt. This third realm of consideration pertains to the specific environment to which the patients present.

### Care setting factors

The context of the patient’s initial injury can have a significant impact on the course of their treatment and likelihood of a successful outcome. The initial treatment can dictate whether surgeons are considering the duration of warm or cold ischemia in their surgical indications for replantation and is contingent upon the medical providers either in the pre-hospital context or within the initial treating emergency department. The patient’s proximity to a hospital that performs replantation surgery varies greatly and itself can cause a significant delay in treatment. Transfer to a hospital that performs replantation is associated with an increased rate of attempt and survival in the context of thumb amputation injuries [37].

Once a patient is at a hospital that is able to provide replantation surgery, there are further factors that impact the success of the replantation. Individual surgeon proficiency, for example, is a contributor to improved outcomes. Surgeons who perform replantation surgeries relatively infrequently have higher rates of failure compared to those who perform these cases more frequently [38]. Additionally, hospital volume correlates to success; the probability of digit survival is 12% greater in hospitals that perform more than 20 thumb replantations annually compared to those that perform 11 or fewer annually [39]. This presents a dilemma—patients will have superior outcomes when they present to hospitals with surgeons who perform these cases more frequently. However, by concentrating these cases to a small number of tertiary hospitals, trainees and attending surgeons elsewhere are deprived of opportunities to gain experience in performing these surgeries. Furthermore, nursing staff do not broadly gain the experience of specialized knowledge in how to care for these patients.

Although this suggests that high-volume hospitals or those that are designated as part of the American Society for Surgery of the Hand/American College of Surgeons hand trauma network should have better outcomes, data are not consistently promising. One participating institution’s report on their results found a 32.9% success rate overall performing 101 hand and digit replantations over the course of 17 years studied [40]. This is in contrast to data suggesting that an annual hospital volume of three replants corresponds to a success rate of 70% [15]. Although the hand trauma network may be a potential solution to ensuring patients have access to care centers with good outcomes, this is not consistently assured.

Several components of the operative management have an influence on the success of replantation attempts. The individual surgeon’s proficiency has a large effect on outcomes following replantation. A replantation designated hospital needs to have more than one proficient surgeon, however; without redundancy in the call structure there can be increased pressure on a limited number of personnel to perform these procedures that often result in disruption to a surgeon’s schedule the following day. There have been suggestions to improve this disruption. Some surgeons advocate for a delayed approach in which amputated digits with limited cold ischemia time that arrive at the hospital in the evening are kept refrigerated and replanted the following morning. In one study, the delayed replantation cohort was found to have equivalent survival and functional outcomes compared to the immediate replantation cohort [41].

The personnel in the operating room is another variable that contributes to the outcome of a replantation attempt. Having other available surgeons allows for a two-team approach which can both expedite surgery and contribute to improved proficiency of participating surgeons by increasing the opportunities to gain skills and experience. The team of anesthesiologists, nurses, and surgical technologists also influences outcome.

The care context is therefore perhaps the most important but simultaneously the least studied factor influencing the outcome of a patient presenting for replantation. This too should be considered when conceiving of indications and contraindications for replantation. Patients treated in Asia, on average, have better outcomes. Within the USA, patients presenting to high-volume replantation centers fare better than those who are treated at hospitals that infrequently perform replantation. An individual surgeon’s replantation volume and proficiency influence success as well. The availability of peri-operative resources including ready operating rooms, equipment, instruments, microsuture, and staff familiar with these items has a critical bearing on success. Anesthesia providers facile with peripheral nerve blocks and normotensive anesthesia who communicate well with the surgical team (and surgical teams who recognize the importance of this communication) enhance the likelihood for good outcomes, as well as the willingness of surgeons to perform these operations more routinely. Negative predictive factors for good outcomes are when a surgical procedure is seen as non-routine, disruptive, and frequently unsuccessful by the surgeon, operating room staff, emergency room personnel, and nursing staff; if excellent outcomes are seen as unattainable by all involved, that is certain to be the case.

When considering whether to indicate a patient for replantation surgery, one must consider long-term outcomes in their rubric of what determines success. The choices made by a surgeon in terms of the osteosynthesis, tendon repair, and nerve repair that all influence the likelihood of digit survival and good functional outcome. The greatest predictor of bony nonunion is post fixation fracture site gap [42]. Reported rates of nonunion following replantation vary widely but have been reported as high as 31% [43]. Bone shortening can be useful in providing better bone-bone contact. It can also limit or eliminate the need for vascular and nerve grafts. The means of fixation that is expedient, allows for stable fixation with reduction in fracture site gap, and requires minimal soft tissue stripping is best.

The outcomes of tendon repairs can be inhibited by restrictive post-operative protocols. Early mobilization (within 14 days of injury) is contingent upon stable bony fixation and strong tendon repairs and is associated with significantly improved TAM. Successful outcomes from nerve repair require a tension-free approximation which may require the use of a graft. Rates of return of two-point discrimination (2PD) following replantation vary but are less than what can be expected in an isolated nerve injury that is repaired primarily or via grafting [44]. In very distal amputations, patients experience an average of 7 mm 2PD regardless of whether a nerve is repaired [45].

Further pertinent variables when considering the predicted success of a replantation surgery include post-operative monitoring and ability to intervene in the setting of vascular compromise. One review found that only 9% of replanted digits that experience vascular compromise are successfully salvaged. The authors concluded that it is cost-ineffective to have continued inpatient monitoring beyond 24 h for single digit (including thumb) replantation and 48 h for multiple digit replantation [46]. Other studies suggest that there may be higher rates of salvage at more prolonged time points via non-operative means (e.g., leech therapy) which could justify longer hospitalization, although this may not be cost-effective [47].

The post-operative protocols for patients undergoing replantation vary widely, and there is no consensus on what protocol is best [48]. There is good evidence that nicotine avoidance and peripheral nerve blockage are associated with improved outcomes. The data on anticoagulation, however, are conflicting with some studies demonstrating improved outcomes with use of antithrombic drugs such as heparin and others finding no difference [49, 50]. Some centers choose to use aspirin plus heparin (either unfractionated or low molecular weight); others use neither. Some centers advocate direct warming of the replanted digit; others promote simply maintaining a warm room citing concerns for thermal injury to the insensate replanted digit. Some centers advocate routine use of anxiolytics to diminish risk of vasospasm; others do not consider this at all. Given the diversity of protocols, it remains unknown what components of the post-operative care of these patients are most critical to success.

## Conclusion

Successful replantation of an amputated part of the upper extremity represents a tremendous challenge for the patient, surgeon, and care setting. Numerous factors pertinent to these three dimensions interact to produce outcomes that range from extremely successful to profoundly disappointing. Knowing when to indicate a patient for replantation versus amputation is far more complex than adhering to a simplified list of absolute and relative indications and contraindications. The cases which land definitively on either end of the amputation/replantation spectrum are rare. The evidence supporting a surgeon’s decision is sparse in areas and often contradictory. Outcome measures for replantation have historically not encompassed the spectrum of the patient’s experience of what defines success in the immediate and long-term period. More recent literature has advocated a stronger focus on this perspective, which may help surgeons decide for whom a replantation can be reasonably expected to provide a good outcome. Within the USA, attempts at replantation and success rates have fallen. This is not a global phenomenon, and this discrepancy merits further study to ensure patients have the opportunity to experience the benefits of this surgery regardless of their geography.

Patient factors relevant for consideration when assessing candidacy for replantation include age and general health, as well as attitude, lifestyle, and cultural considerations. In the first report of two cases of upper extremity replantation, Drs. Malt and McKhann presciently write:

The intangibles are more difficult to specify. Is the damage such that replantation can reasonably be expected to give a better functional result than a prosthesis? Do the patient’s age, health, occupation, and economic circumstances suggest that a long convalescence with the hope of improved performance would better serve the patient than would an early fitting of an artificial limb and prompt vocational rehabilitation? Is the patient one who can bear hospitalization and multiple operations with equanimity or will he become a psychic cripple? All these considerations must be weight by the surgeon before he attempts to reunite a limb to the body. [1]

These words remain true, and such “intangibles” must be considered when counseling a patient regarding what treatment will provide the best outcome. It is also essential for the surgeon to honestly convey what can be anticipated post-operatively in terms of hospital stay, occupational therapy, need for revision surgery, and functional outcome.

Injury factors will influence this discussion as well. Sharp injuries fare better than crush and avulsion injuries. The amount of contamination, tissue loss, and level of amputation further influence the likelihood of achieving a perfused, sensate, and dextrous hand that is useful to the patient in their everyday life. Concomitant injuries to the ipsilateral extremity or to the rest of the patient may impede the ability to achieve such an outcome. Life-threatening injuries may preclude an immediate replantation attempt and require a surgeon to rely on alternative means of delayed replantation such as ectopic banking.

Success in replantation requires careful consideration of whom to indicate for surgery contingent on rapidly acquiring an understanding of the patient and injury factors in a time-pressured environment where a patient-provider relationship is first being established. This is then followed by having an enthusiastic and skilled surgeon perform efficient yet meticulous surgery comprising many steps using specialized equipment, often in the middle of the night. Success is not yet guaranteed when a doppler signal is located on a digit, or when the patient makes it to the ICU, or even at the time of discharge from the hospital. The patient must then be shepherded through their recovery and rehabilitation, encouraged to participate in therapy, provided the resources necessary to optimize success, and advised of when they require additional procedures when their progress has plateaued. These patients deserve to be followed for years after their replantation to determine how their sensation and motion progress and to elicit their perspective on whether the replanted part is useful in their lives. This allows a surgeon to evaluate functional outcomes quantitatively and from the patient subjectively, not only to inform quality improvement and technical refinement but also to contribute to an evolving understanding of best practices in achieving superior outcomes.
